# A New Method to Overcome Carboxyamide Formation During AFEX Pretreatment of Lignocellulosic Biomass

**DOI:** 10.3389/fchem.2021.826625

**Published:** 2022-01-21

**Authors:** Hui Dong, Leonardo da Costa Sousa, Bryan Ubanwa, A. Daniel Jones, Venkatesh Balan

**Affiliations:** ^1^ Department of Chemical Engineering and Material Science, Michigan State University, Lansing, MI, United States; ^2^ Department of Engineering Technology, College of Technology, University of Houston, Sugarland, TX, United States; ^3^ Great Lakes Bioenergy Center, Michigan State University, East Lansing, MI, United States; ^4^ Department of Biochemistry and Molecular Biology, Michigan State University, East Lansing, MI, United States

**Keywords:** lignocellulosic biomass, alkaline pretreatment, AFEX process, acetamide, animal feed, ester linkages

## Abstract

Lignin-carbohydrate complexes (LCCs) in the plant cell wall are responsible for providing resistance against biomass-degrading enzymes produced by microorganisms. Four major types of lignin-carbohydrate bonds are reported in the literature, namely, benzyl ethers, benzyl esters, phenyl glycosides, and acetyl ester linkages. Ester’s linkages in the plant cell wall are labile to alkaline pretreatments, such as ammonia fiber expansion (AFEX), which uses liquid or gaseous ammonia to cleave those linkages in the plant cell wall and reduce biomass recalcitrance. Two competing reactions, notably hydrolysis and ammonolysis, take place during AFEX pretreatment process, producing different aliphatic and aromatic acids, as well as their amide counterparts. AFEX pretreated grasses and agricultural residues are known to increase conversion of biomass to sugars by four- to five-fold when subjected to commercial enzyme hydrolysis, yielding a sustainable feedstock for producing biofuels, biomaterials, and animal feed. Animal feed trials on dairy cows have demonstrated a 27% increase in milk production when compared to a control feedstock. However, the presence of carboxamides in feedstocks could promote neurotoxicity in animals if consumed beyond a certain concentration. Thus, there is the need to overcome regulatory hurdles associated with commercializing AFEX pretreated biomass as animal feed in the United States. This manuscript demonstrates a modified pretreatment for increasing the digestibility of industrial byproducts such as Brewer’s spent grains (BSG) and high-fiber meal (HFM) produced from BSG and dry distillers grains with soluble (DDGS), while avoiding the production of carboxamides. The three industrial byproducts were first treated with calculated amounts of alkali such as NaOH, Ca(OH)_2_, or KOH followed by AFEX pretreatment. We found that 4% alkali was able to de-esterify BSG and DDGS more efficiently than using 2% alkali at both 10 and 20% solids loading. AFEX pretreatment of de-esterified BSG, HFM, and DDGS produced twofold higher glucan conversion than respective untreated biomass. This new discovery can help overcome potential regulatory issues associated with the presence of carboxamides in ammonia-pretreated animal feeds and is expected to benefit several farmers around the world.

## Introduction

Industrial lignocellulosic biomass byproducts such as Brewer’s spent grains (BSG) and high-fiber meal (HFM) produced in distilleries and dry distillers grains and soluble (DDGS) produced in corn ethanol plant are sustainable feedstocks for producing fuels, chemicals, biomaterials, and animal feed. Both BSG and DDGS are extensively used as ruminant animal feed. BSG contain a significant quantity of Tannins that are reported to interfere in the absorption of essential nutrients, amino acids, and digestive enzymes, thereby reducing feed utilization (Liu, 2011; Westendorf et al., 2014; Oryschak et al., 2010a; Oryschak et al., 2010b; Choct et al., 2010; Lacassagn et al., 1998). As the quantity of BSG in the diet increased, the feed conversion ratio decreased (Alabi et al., 2014; Ashour et al., 2019). Extensively using lignocellulosic biomass as animal feed will help displace grains, such as corn, soybean, and sorghum, and reduce global hunger (Salami et al., 2019). Lignocellulosic biomass recalcitrance has been attributed to high lignin content, acetylated hemicellulose, and highly crystalline cellulose (Marriott et al., 2016). Hence, using untreated lignocellulosic biomass as feedstock provides low metabolizable energy to animals. Four types of chemical linkages are reported in the literature between lignin and hemicellulose that form a lignin-carbohydrate complex (LCC). They include benzyl ethers, benzyl esters, phenyl glycoside, and acetyl linkages (Balakshin et al., 2011). Some of the ester linkages include monomeric *p*-coumarate (pCA) and ferulate-linkages on lignin, glucuronoarabinoxylan (GAX), eight de-hydro-diferulic acids (DFA) resulting from five possible coupling modes (8-5′-, 8-O-4′-, 8-8′-, 5-5′-, and 4-O-5′-coupling) connecting glucuronoarabinoxylan (GAX) and GAX-hemicellulose sugar polymer, monomeric ferulate connecting lignin to GAX, and acetylated sugars (Hatfield et al., 1999; Bunzel et al., 2004; Mnich et al., 2020). Particularly, pCA is mostly acylated to the lignin polymer at the γ-carbon of the side chain region, particularly in syringyl moieties and to a lesser extent on arabinosyl side chains of arabinoxylan (Hatfield et al., 2008). These linkages make the lignocellulosic biomass highly recalcitrant and less digestible in ruminant animals.

Several acids- and alkali-catalyzed pretreatment methods are widely used to produce highly digestible ruminant animal feed (Falls et al., 2017; Zhao et al., 2018; Liang et al., 2020). Chemical catalyzed pretreatment processes help open the forage plant cell wall, reduce the recalcitrance, and improve the digestibility of the feed. Some of the prominent pretreatments include dilute acid, NaOH or KOH catalyzed H_2_O_2_, ionic liquids, dilute acid or base catalyzed steam explosion, liquid hot water, CO_2_ explosion, organosolv, ammonia percolation, and lime pretreatment (Baruah et al., 2018). However, most of the catalyst used in pretreatment process is miscible in water and get embedded into the biomass, requiring expensive processing step to remove or neutralize them after pretreatment. Ammonia fiber expansion (AFEX) is one of the leading alkaline pretreatments processes that uses volatile ammonia as catalyst and up to 97% of ammonia can be recycled and used for subsequent processing cycle (da Costa Sousa et al., 2009; Balan et al., 2009a; Balan et al., 2011; Chundawat et al., 2011; da Costa Sousa et al., 2016; Balan et al., 2017; Bals et al., 2019; Mor et al., 2019; O Ribeiro et al., 2020; Xu et al., 2020). AFEX pretreatment of various feedstocks such as rice and wheat straw, corn stover, and oat hull followed by densification and drying helped produce dry biomass pellets (<10% moisture) with increased bulk density (588–634 kg/m^3^) like shelled corn grains (720 kg/m^3^) and offered the advantage to transportation and store biomass in grain silos (Hoover et al., 2014). Microorganisms in ruminant animals (dairy and beef cows, sheep, goats, buffalo, horse, donkey, mule, and camels) in the gut are capable of efficiently hydrolyzing the cellulose and hemicellulose in the AFEX pretreated lignocellulosic biomass and provide energy to animals (Mokomele et al., 2018). Some of the recently concluded AFEX pretreated densified wheat straw as animal feed trials, especially dairy cows and buffaloes has demonstrated increased metabolizable energy and increased milk production as high as 27% (Mor et al., 2018; Beauchemin et al., 2019; O Ribeiro et al., 2020). Pretreatments of monocot (e.g., corn stover, forage sorghum, rice straw, sugarcane bagasse, and perennial grasses such as miscanthus) and dicot (e.g., poplar hard wood, agave, and alfaalfa) feed stocks using AFEX have also shown higher sugar conversion like AFEX-treated corn stover when subjected to commercial biomass-degrading enzyme hydrolysis and are expected to be excellent animal feed (Balan et al., 2011; Balan et al., 2012) (Table 1).

**TABLE 1:** Sugar conversion of lignocellulosic biomass before and after AFEX pretreatment.

Lignocellulosic biomass	AFEX condition^a^	Untreated^b^	AFEX^b^	Enzyme hydrolysis/g of glucan^c^
Corn stover	90°C, 1:1, 60%, 5 min	32%(G), 10% (X)	95%(G), 70% (X)	Cellulase 33 mg, β-glucosidase 33 mg, and xylanase 3.3 mg
Switch grass	100°C, 1:1, 80%, 5 min	16%(G), 3% (X)	93%(G), 70% (X)	Cellulase 33 mg, β-glucosidase 22 mg
Sugarcane bagasse	140°C, 2; 1, 150%, 30 min	20%(G), 18% (X)	90%(G), 65% (X)	Cellulase 33 mg, β-glucosidase 33 mg, and xylanase 8 mg
Sugarcane leaves	100°C, 1:1 60%, 30 min	20%(G), 18% (X)	90%(G), 65% (X)	Cellulase 33 mg, 33 mg β-glucosidase, and xylanase 15 mg
Forage sorghum	140°C, 2:1, 120%, 5 min	32%(G), 15% (X)	90%(G), 95% (X)	Cellulase 33 mg, β-glucosidase 17 mg, and xylanase 15.6 mg
Miscanthus	140°C, 2:1, 233%, 30 min	10%(G), 6% (X)	84%(G), 85% (X)	Cellulase 33 mg, β-glucosidase 33 mg, and xylanase 6 mg
DDGS	90°C, 1:1, 40%, 5 min	63%(G), 6% (X)	85%(G), 81% (X)	Cellulase (15 FPU/g of cellulose), β-glucosidase (40 IU/g of cellulose), pectinase (50 IU g DDGS dwb), and feruloyl esterase (2 IU/g of DDGS)
Alfaalfa	85°C, 2:1, 30%, 5 min	38%(G), 34% (X)	68%(G), 86% (X)	Cellulase 5 FPU/g of dry matter
Poplar	180°C, 2:1, 233%, 30 min	5%(G), 5% (X)	93%(G), 65% (X)	Cellulase 125 mg, β-glucosidase 33 mg, and xylanase 125 mg
Rice straw	140°C, 1:1, 80%, 30 min	20%(G), 10% (X)	85%(G), 86% (X)	Cellulase 33 mg, β-glucosidase 33 mg, xylanase 2.7 mg, and pectinase 3.7 mg
Reed canary grass	100°C, 0.8:1, 60%, 30 min	20%(G), 0% (X)	89%(G), 81% (X)	Cellulase 33 mg, β-glucosidase 33 mg, and xylanase 8 mg
Elephant grass	90°C, 1:1, 60%, 5 min	18%(G), 10% (X)	83%(G), 51% (X)	Cellulase 5 IU/g of dry matter, β-Glucosidase 5.7 U
Agave bagasse	120°C, 2:1, 40%, 30 min	20%(G), 10% (X)	80%(G), 68% (X)	Cellulase 20 mg (Ctec3)
Agave leaves	100°C, 3:1, 20%, 30 min	15%(G), 5% (X)	80%(G), 50% (X)	Cellulase 20 mg (Ctec3)

a Pretreatment conditions are given in the following order: temperature, ammonia to biomass loading, moisture (dry weight basis), and residence time.

b Sugar conversions are greatly influenced by the enzyme activities present in the cocktail. Here, G, glucan; X, xylan conversion.

c Hydrolysis was carried out at 1% glucan loading for 168 h.

The majority of the ester linkages in lignocellulosic biomass are cleaved by ammonia catalyst during AFEX pretreatment (Balan et al., 2009b). Two competing reactions take place: 1) hydroxide ion (generated from water) attacks ester linkages to produce hydrolysis products such as acetic acid, ferulic acid, and coumaric acid and several di-ferulate di-acids and 2) ammonia attacks ester linkages to produce ammonolysis products, for example, monoamides such as acetamide, feruloylamide, coumaroyl amide, and several di-ferulate mono- and di-amides (Chundawat et al., 2010; Vismeh et al., 2018; Chundawat et al., 2020).

R-CO-OR’ + OH^−^ R’OH + R-CO-O^−^ (hydrolysis reaction)

R-CO-OR’ + NH_3_ R’OH + R-CO-NH_2_ (ammonolysis reaction)

Similar cleavages of ester linkages are reported when lignocellulosic biomass is pretreated using dilute acid or dilute alkali (Chen et al., 2012; Saulnier et al., 2020). The carboxamides produced during ammonia pretreatment of forages are considered as potential feed carcinogens when consumed beyond a certain concentration, and there is widespread fear of these compounds entering into the human food chain (Chundawat et al., 2010; Vismeh et al., 2018 Mokomele et al., 2018; Bals et al., 2019; Moore et al., 2019). For example, acetamide (CAS60-35-5) is classified as one of the possible neurotoxins (IARC Casa Grop-2B) following chronic exposures at high dosage. *In vitro* lab studies have shown that the amides are reported to inhibit microorganisms such as yeast (Tang et al., 2015; Xue et al., 2018). Literature reports show that acetamide is generally of low acute oral toxicity in rodents. However, high doses of acetamide affect the central nervous system, liver, kidneys, spleen, and lungs (Moore et al., 2019; Nault et al., 2020).

To overcome the formation of carboxamides during AFEX pretreatment, we have developed a two-step pretreatment process for the first time that will help overcome the bottleneck of using them as animal feed (Balan and Sousa, 2019). Three different industrial lignocellulosic biomass byproducts, namely, BSG, HFM, and DDGS have been used in this study. We, first, calculated the amount of alkali such as NaOH and Ca(OH)_2_ that are needed to de-esterify ester linkages in lignocellulosic biomass. The right quantity of alkali is mixed with water, evenly added to lignocellulosic biomass, and heated up to 100°C for a fixed residence time. The presence of alkali solution on biomass facilitated the cleavage of ester linkages to produce corresponding organic acids. Subsequently, ammonia was loaded to the de-esterified biomass with appropriate moisture in a high-pressure reactor to carry out the AFEX process. Ammonia helps catalyze the cleavage of LCCs and solubilize larger portions of lignin and hemicelluloses, which are displaced to the surface of biomass when ammonia is released from the high-pressure reactor (Balan et al., 2009b; Chundawat et al., 2011). The two-step pretreatment process for BSG, HFM, and DDGS yielded no significant increase in carboxamide content relative to the untreated materials and showed a comparable digestibility to that of the one-step AFEX pretreated biomass when subjected to commercial biomass-degrading enzymes. This modified pretreatment procedure will help produce highly digestible, safe, and sustainable animal feed, benefiting several farmers around the world.

## Materials and Methods

### Sources of Biomass and Composition Analysis

The DDGS was collected from Big River Resources, LLC (West Burlington, IA), and stored at ambient temperature until it was used. The composition of DDGS biomass on a dry weight basis was determined using the NREL protocol (Sluiter et al., 2010). It contains glucan (∼21%), xylan (∼8.2%), and protein (∼25.6%). The BSG was obtained from Anheuser-Busch Inc. Brewery Plant St. Louis, MO, with the help of our industrial partner Trucent, Dexter, MI. BSC was oven-dried at 60°C until the moisture content was less than 8% and stored at room temperature. The composition of BSG on a dry weight basis was found to contain glucan (14.7%), xylan (16.8%), arabinan (7%), and protein (34.3%) using the NREL protocol. High-fiber meal (HFM) was separated from BSG using the reported protocol (He et al., 2019). Briefly, the method entailed using BSG at 5% (w/w) solids was mixed with pure water and incubated at 60°C for 4 h to solubilize protein. Then, the slurry was transferred to a sieve shaker to separate the fiber from soluble protein. The retentate, the fibrous portion of BSG left behind on the sieve, is called HFM and dried at 60°C in a convection oven for 24 h until the moisture content was less than 8% and stored at room temperature. The composition of HFM on a dry weight basis was found to contain glucan (18.1%), xylan (23.8%), arabinan (9.6%), and protein (18.3%).

### AFEX Pretreatment

AFEX pretreatment was carried out using previously reported protocol without any extraction (Dale et al., 1985; Balan et al., 2009a). The moisture content of BSG/HFM/DDGS was increased to 40% by spraying water, followed by loading into a high-pressure tubular reactor. The moisture content of the samples was measured using an A&D moisture analyzer fitted with an IR lamp. The reactor was first closed and degassed to remove any air from the reactor, and a known amount of liquid ammonia was loaded to BSG or DDGS at 1:1 ratio and headed by an external heater until the temperature in the reactor reached 100°C. Once the desired temperature was reached, the pretreatment was continued for another 30 min, before the ammonia was vented from the top. AFEX treated samples were transferred to aluminum trays and dried in a fume hood overnight to remove residual ammonia and moisture. The samples were then stored in zip-lock bags in a refrigerator. The total nitrogen content of the biomass was measured by the Kjeldahl method using the NREL protocol (Hames et al., 2008).

### Ammonium Hydroxide Pretreatment

BSG, HFM, and DDGS was pretreated using 3% aqueous ammonium hydroxide at 9% solids loading for 6 h at 60°C (Dien et al., 2013; Le et al., 2021). Then, the pretreated biomass was filtered using the Buchner funnel, and the sample was dried in the fume hood overnight to remove residual ammonia.

### Commercial Biomass-Degrading Enzyme Hydrolysis

To simulate biomass digestibility experiments by rumen in the gut, we carried out enzymatic hydrolysis at 3% solids loading. The total reaction volume was 50 ml, and experiments were carried out in a 125 ml flask (Mokomele et al., 2018). Experiments were carried out in an orbital shaker incubator set at 200 rpm at 50°C for 24 h. About 3 mg of commercial enzyme cocktail was added in the ratios 80:15:5 (Ctec2: Htec2: Pectinex). All the commercial enzymes used in this experiment were provided by Novozymes. About 1 ml of hydrolyzed samples was heated at 100°C for 10 min, cooled down on ice, and passed through a 0.2 micro syringe nylon filter before analyzing the sample using HPLC using Biorad Aminex 80P column to estimate the glucose and xylose conversion (Kim et al., 2008). Mixed sugar comprising glucose and xylose at varying concentration was used to construct the standard curve (Ruiz and Ehrman 1996).

### De-Esterification of BSG and DDGS Using Different Alkali

In this study, de-esterification of BSG or DDGS was carried out using mild alkali such as Ca(OH)_2_ and strong alkali such as sodium hydroxide (NaOH). The alkali concentration was varied between 2 and 4%, while the DDGS or BSG solid loadings were maintained as 10 or 20%. The samples were heated at 80°C for 15 min and filtered using a mirror cloth. The alkali-treated BSG, HFM, and BSG samples were dried in the hood at room temperature to reduce the moisture content to 60%. The de-esterified biomass was subjected to AFEX pretreatment using 1:1 ammonia to biomass loading, 100°C for 15 min residence time. The pretreated biomass was collected in the tray and kept in the hood overnight until most of residual ammonia is removed.

### Acetamide Measurement in Pretreated Biomass

Acetamide was quantified using an established protocol using GC-MS without any derivatization (Vismeh et al., 2018). Briefly, acetamide was extracted using water as a solvent in an ASE 200 Accelerated Solvent Extractor (Dionex) at approximately 11 ml of water per gram sample for two cycles. About 1.0 µL of extracts of untreated and pretreated lignocellulose biomass (HFM, BSG, DDGS) was injected into a DB-WAX column (Agilent, Santa Clara, CA) with dimension 30 × 0.25 mm i.d., 0.25 μm using helium as carrier gas at 1.5 ml/min and injector temperature of 240°C. Mass spectra (70 eV electron ionization) were collected in full scan mode (*m/z* 30–400). The column temperature was programmed as follows: initial column temperature at 50°C and holding for 1 min; and increasing to 250°C at 15°C/min and holding for 2 min. The total run time for each sample was 16.3 min/sample. The analytes were quantified using extracted ion chromatograms of molecular ions *m/z* 59 and 62 for acetamide and acetamide-*d*_3_ (internal standard), respectively.

## Results and Discussion

### Lignocellulose Byproducts From Industry and Value Addition

**FIGURE 1:**
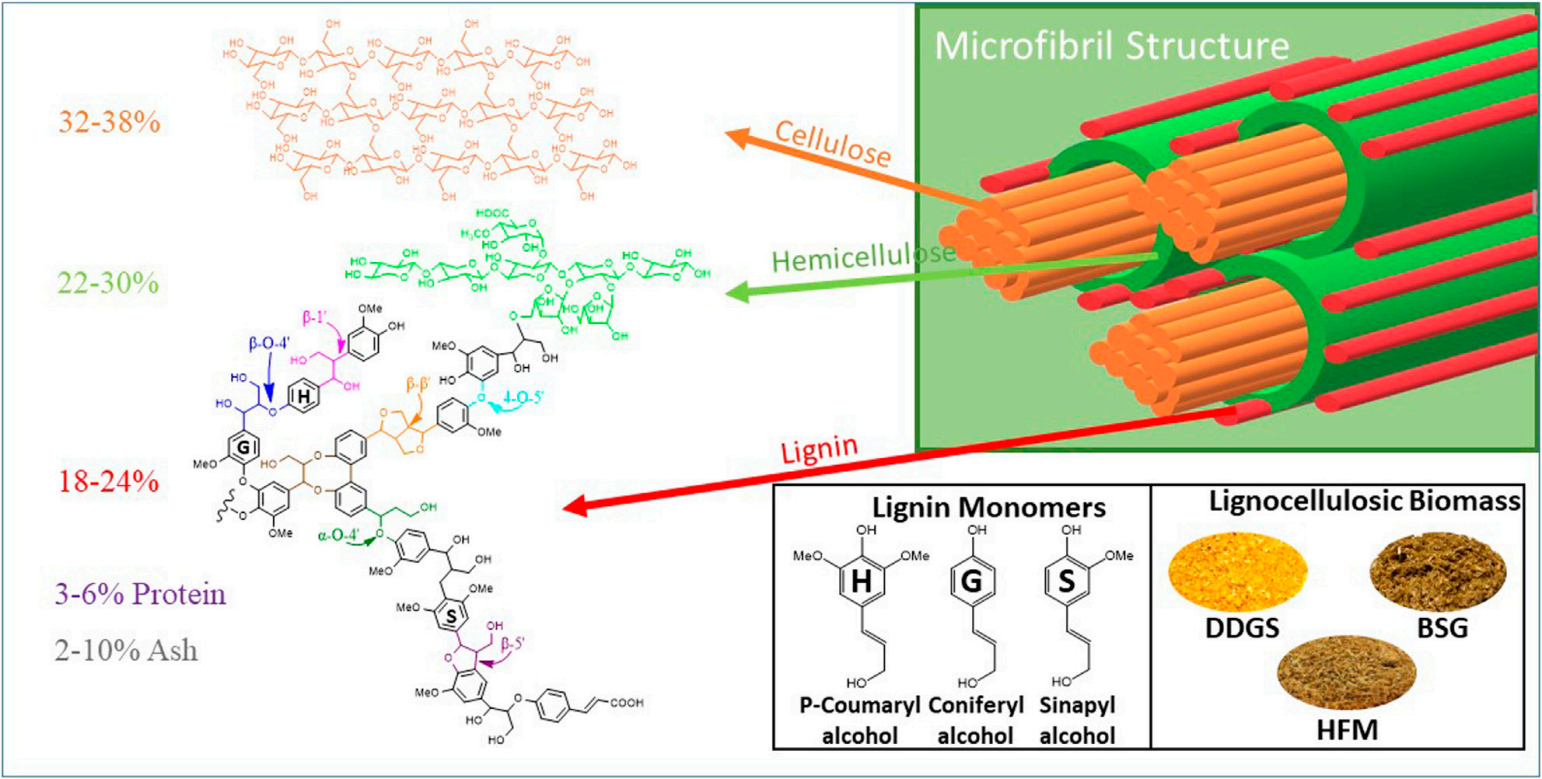
Ultrastructure of plant cell wall comprising cellulose, hemicellulose, lignin, protein, and ash in different ratios. Detailed structure of lignin monomers. Three different types of lignocellulosic biomass, namely, dry distillers grains with soluble (DDGS), high-fiber meal (HFM), and Brewer’s spent grains (BSG), used to produce de-esterified biomass without carboxamides are given.

Most industrial lignocellulosic byproducts are comprised of cellulose, hemicellulose, lignin, and protein. The composition of these materials depends on how the feedstocks are processed. Naturally, these lignocellulosic biomasses are recalcitrant due to the complex network of different components and crystallinity of cellulose (Figure 1). BSG is a byproduct stream of the beer brewing process and comprises the extracted residue of barley malt alone or mixed with other cereal grains (Westendorf and Wohlt, 2002). Modern large-scale breweries generate approximately 20 kg of wet BSG per hectoliter of beer. A large-scale North American brewery will produce over 100,000 tons of wet BSG per year, and more than three million tons of BSG/year are available in the US alone. Wet BSG coming out of the brewery has a short shelf life and is therefore immediately sold to nearby dairy or cattle farms. Simply drying the BSG will increase the shelf life and make it suitable for sale in more distant markets, but the drying process is expensive (Aliyu and Bala, 2011). Therefore, the brewing industry seeks means to enhance the value of BSG beyond just a dried product. It is posited that enhanced value can be achieved by creating nutritionally rich fractions from the BSG, which are then sold into targeted species and life cycle-specific markets.

BSG can be mechanically separated into a high protein meal (HPM) and a high-fiber meal (HFM), representing approximately 35 and 65% of BSG dry matter, respectively (Kishi et al., 1991). Preliminary economic analysis shows that dry HPM and HFM can give incremental profit improvement relative to wet BSG (Buffington 2014). However, the economics can be made even more compelling by further upgrading BSG or its fractions for specific animal nutrition markets.

Several past research and review articles provide a wealth of knowledge about BSG composition, feeding trials and nutrition value to ruminant and non-ruminant animals, and new methods of upgrading BSG into higher-value products, including human food and protein concentrate (Mussatto et al., 2006; Celus et al., 2009; Lynch et al., 2016; Chetrariu and Dabija 2020; Kavalopoulos et al., 2021; Marcus and Fox 2021). BSG also have sizable lignin (cross-linked phenolic polymers) and tannins (with basic units like gallic acid and flavone). The energy content of BSG varied from different sources and types of grain used in a particular brewery. Some of the animal nutrition parameters for BSG include total digestible nutrients (TDN), 71–76%; net energy for lactation (NEL), 0.73–0.82 mcal/lb; net energy for maintenance (NEM), 0.79–0.88 mcal/lb; and net energy for gain (NEG) 0.51–0.58 mcal/lb (Santosa et al., 2003). Feeding wet BSG to animals increased the risk of mycotoxins in their diet, while heat processing was proven to degrade mycotoxin (Schwarz et al., 2018). BSG amino acid profile analysis showed that lysine and threonine are the first two limiting amino acids. High temperature (>190°C) used during dilute acid or alkali-catalyzed steam explosion producing undesirable inhibitory products that inhibit microbes during the digestion process also affects the solubility and stability of proteins (Kemppainen et al., 2016). Adding feed enzymes such as xylanase increased the nutrient availability to animals (Beauchemin et al., 2003; Forssell et al., 2008; Treimo et al., 2008; Denstadli et al., 2010).

DDGS is another co-product produced in the corn ethanol plant 22 million tons in 2019 in the United States and is widely used around the world as animal feed. Prior studies have shown that DDGS digestibility could be improved with AFEX and hot water treatment (Kim et al., 2008). Digestibility of pretreated DDGS could be further improved when supplemented with hemicellulase enzymes such as pectinase and xylanase (Dien et al., 2008; 2013). Pretreating DDGS using dilute acid followed by enzyme hydrolysis and digested the fibers, thereby enriching the protein content, enhanced digestibility and metabolizable energy (Fries-Craft and Bobeck, 2019; Buenavista et al., 2021).

### Ammonia Pretreatment

Traditionally, farmers have performed an ammoniation process in the field by pumping anhydrous ammonia under a tarp containing bales of hay at 3–4g/100 g dry biomass, at ambient temperature for 6–8 weeks, to improve the digestibility by 10–20% of late harvest grasses or straw (Solaiman et al., 1979). By doing this, the crude protein of the hay increases by 5–10% due to ammonia reacting with biomass. The in-field ammoniation process provides a modest increase in digestibility but is a safety concern and is expensive as the ammonia is not recoverable. It was not until 1982 that a contained reactor process involving pressurized, high-temperature, concentrated ammonia (>30%) and known as ammonia fiber expansion or AFEX was developed to increase the enzymatic digestibility of biomass (Balan et al., 2009a) by opening the cell wall. DuPont also developed a low-concentration aqueous ammonia (<15% NH_4_OH) process to pretreat biomass for the production of bio-ethanol in their commercial plant in Nevada, Iowa (Dunson et. al., 2007). Since ammonia is a volatile alkali, it offers the unique advantage of evaporation-based separation and recovery (>98%) after treating the biomass. Conventional AFEX processing uses liquid ammonia (0.3–2 g NH_3_/g dry biomass) with moist biomass (0.1–2 g water/g dry biomass) and heating (40–120°C) for a period of 5–180 min. Several variations to the conventional AFEX process have been discovered, such as using gaseous ammonia (Campbell et al., 2013; Balan et al., 2017), extractive ammonia (EA) which improves ammonia’s contact with the biomass, and recovery of ammonia after treatment (Sousa et al., 2009; Chundawat et al., 2013). The advantages and disadvantages of each of the different ammonia pretreatment processes are given in Table 2.

**TABLE 2:** Different Ammonia pretreatment processes and their advantages/disadvantages.

	Ammonition of biomass in a pit	AFEX (liquid ammonia)	AFEX (gaseous ammonia)	Extractive ammonia
Advantages	Low cost and pretreatment can be carried out on the farm	Produce highly digestible biomass	Energy-efficient process of using ammonia as gas phase and produce highly digestible biomass	Can extract up to 50% of lignin and produce highly digestible cellulose III
Disadvantages	Time-consuming, expensive, ammonia is not recoverable and inefficient process	Need high-pressure reactor, energy-intensive process (due to change of the state of ammonia)	Need high-pressure reactor systems	Need very high pressure, need higher ammonia to biomass loading (6:1), expensive

In the new two-step pretreatment process reported in this manuscript, DDGS, HFM, and BSG were first de-esterified using alkali [Ca(OH)_2_ and NaOH] and filtered and dried until the moisture of the sample was 60%. Subsequently, the de-esterified biomass was subjected to AFEX pretreatment as described in materials and methods section. The two-step pretreatment DDGS, HFM and BSG, looks like the respective AFEX pretreated biomass samples. The following events take place during the two-step pretreatment process: 1) esters cleaved during alkali [Ca(OH)_2_ and NaOH] treatment, 2) most of the lignin-carbohydrate complex (LCC) cleavage and degradation products formation, 3) lignin/hemicellulose got solubilized in ammonia redistribution to the surface when pressure released, and 4) cellulose de-crystallization. Solubilized lignin and hemicellulose are relocated to the surface of the plant cell wall when ammonia is released from the reactor, similar to a previous report (Chundawat et al., 2011). This makes the biomass porous (>10 nm in diameter) and enhances the accessibility of cellulase enzymes.

### Carboxamide Production During AFEX Pretreatment

**FIGURE 2:**
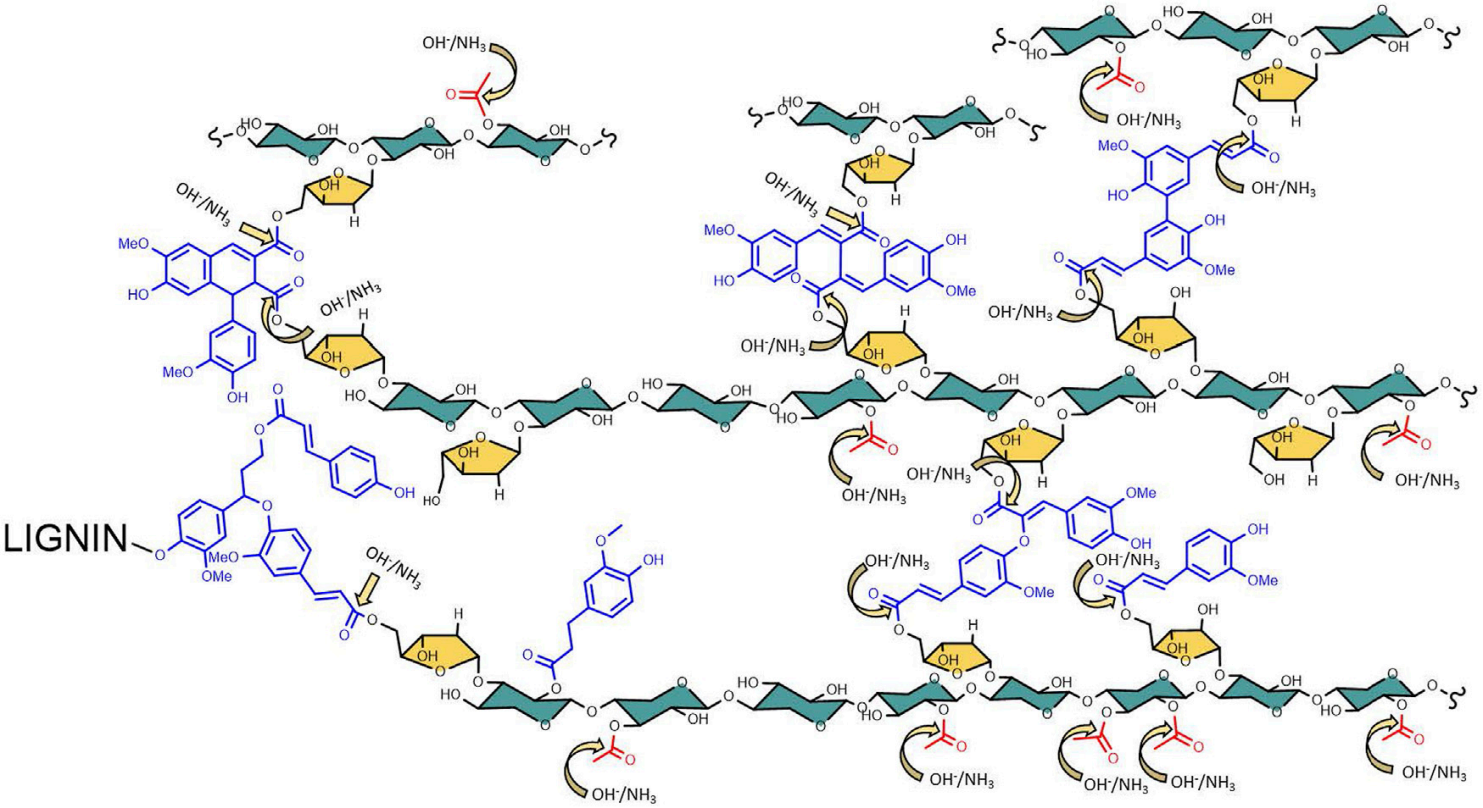
Arabinoxylan lignin linkages in lignocellulosic biomass. During the AFEX process, the targeted ester linkages sites are prone to attach to hydroxide and ammonia, producing the corresponding acids or carboxamide functional groups.

**FIGURE 3:**
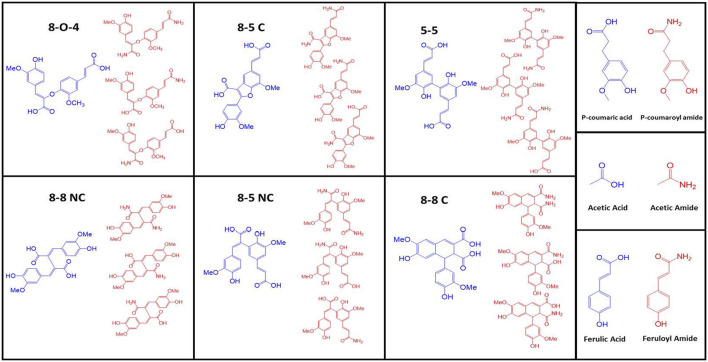
Some of the major degradation products produced during AFEX pretreatment. Here, six different di-ferulate di-acids (8-O-4, 8-5C, 5-5, 8-8 NC, 8-5 NC, 8-8C) are produced when corresponding di-ferulate ester linkages are cleaved by hydroxyl ions (shown in blue color). On the contrary, when ammonium ion cleaves the di-ferulate ester linkages, corresponding di-ferulates with mono- or di-amides are produced (shown in red color). Other degradation products produced during AFEX pretreatment include p-coumaric acid, acetic acid, and ferulic acids (shown in blue) when hydroxyl ion cleaves the corresponding ester linkages. Similarly, p-coumaroyl amide, acetamide and feruloyl amide (shown in red) are produced when ammonia cleaves the corresponding ester linkages.

Most of the lignocellulosic biomass produced by monocot plants have ester linkages such as acetyl, feruloyl, and coumaroyl functional groups (Balan et al., 2009b) (Figure 2). De-esterifying the lignocellulosic biomass is known to improve enzyme digestibility (Chen et al., 2012; Kim et al., 2016). These ester linkages are cleaved when treated with alkali, such as NaOH, KOH, Ca(OH)2, and NH4OH. The cleavage kinetics is influenced by the strength of alkali and the hydrolyzed product dependent on the type of alkali used. For example, NaOH is one of the strongest alkali cleave ester linkages faster than Ca(OH)2. Treating ester linkages using ammonium hydroxide produces corresponding acids and amides (ammonolysis and hydrolysis), while all other alkali produces corresponding organic acids (hydrolysis). One can measure the ester linkages in lignocellulosic biomass by quantifying the organic acids after alkali treatment such as acetic, ferulic, and coumaric acids or their corresponding carboxamides. Based on our previous studies, possible de-esterified products produced after AFEX process are summarized in Figure 3 (Vismeh et al., 2013). Six different di-ferulate di-acids (8-O-4, 8-5C, 5-5, 8-8 NC, 8-5 NC, 8-8C) are produced when corresponding di-ferulate ester linkages are cleaved by hydroxyl ions. On the contrary, when ammonium ion cleaves the di-ferulate ester linkages, 18 different di-ferulates with mono- or di-amides are produced. Other degradation products produced during AFEX pretreatment include *p*-coumaric acid, acetic acid, and ferulic acids when hydroxide ion cleaves the corresponding ester linkages and *p*-coumaroyl amide, acetamide, and feruloylamide (Chundawat et al., 2010; Vismeh et al., 2013).

**FIGURE 4:**
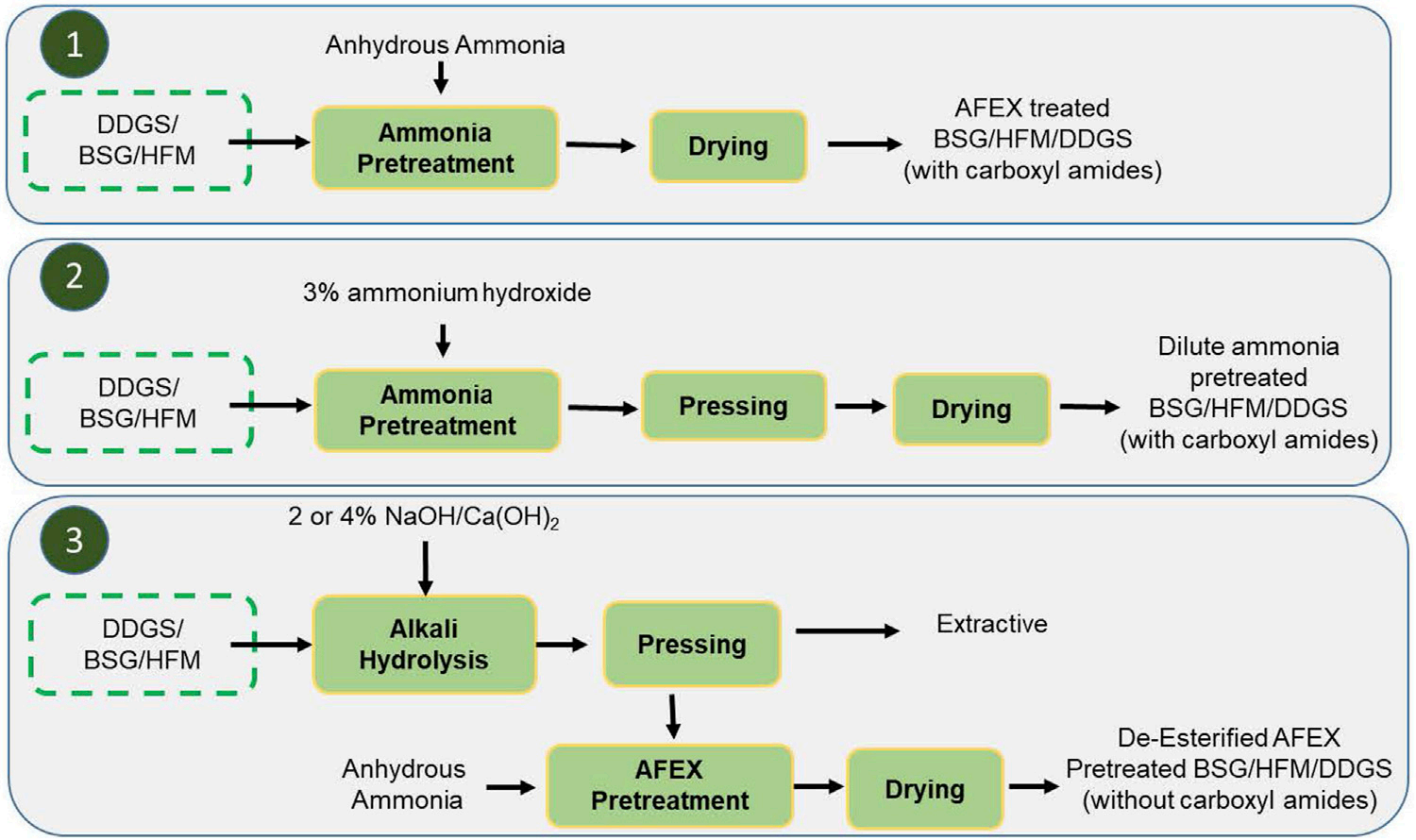
Three different scenarios of alkali treatment of BSG/HFM/BSG. Here, 1, AFEX pretreatment process; 2, dilute ammonium hydroxide pretreatment and 3, either 2 or 4% NaOH, Ca(OH)_2_ pretreatment followed by anhydrous ammonia pretreatment. Scenarios 1 and 2 produce carboxamides in pretreated biomass, while scenario 3 produces pretreated biomass without carboxamides.

In this work, we evaluated three different scenarios: 1) AFEX pretreatment of BSG/HFM/DDGS (using anhydrous liquid ammonia), 2) 3% dilute ammonium hydroxide pretreatment of BSG/HFM/DDGS, and 3) alkali such as NaOH or Ca(OH)_2_ pretreatment at varying concentrations (2 or 4%) followed by AFEX pretreatment. To monitor the cleavage of ester linkages, we analyzed the concentration of acetic acid and acetamide in the pretreated biomass extracts using the established GC-MS method against the respective standard (acetic acids and acetamides). As expected, Scenarios 1 and 2 produced acetamide and acetic acid in pretreated biomass, while scenario 3 produced only acetic acids (Figure 4).

### De-Acetylation and Ammoniation of BSG

**FIGURE 5:**
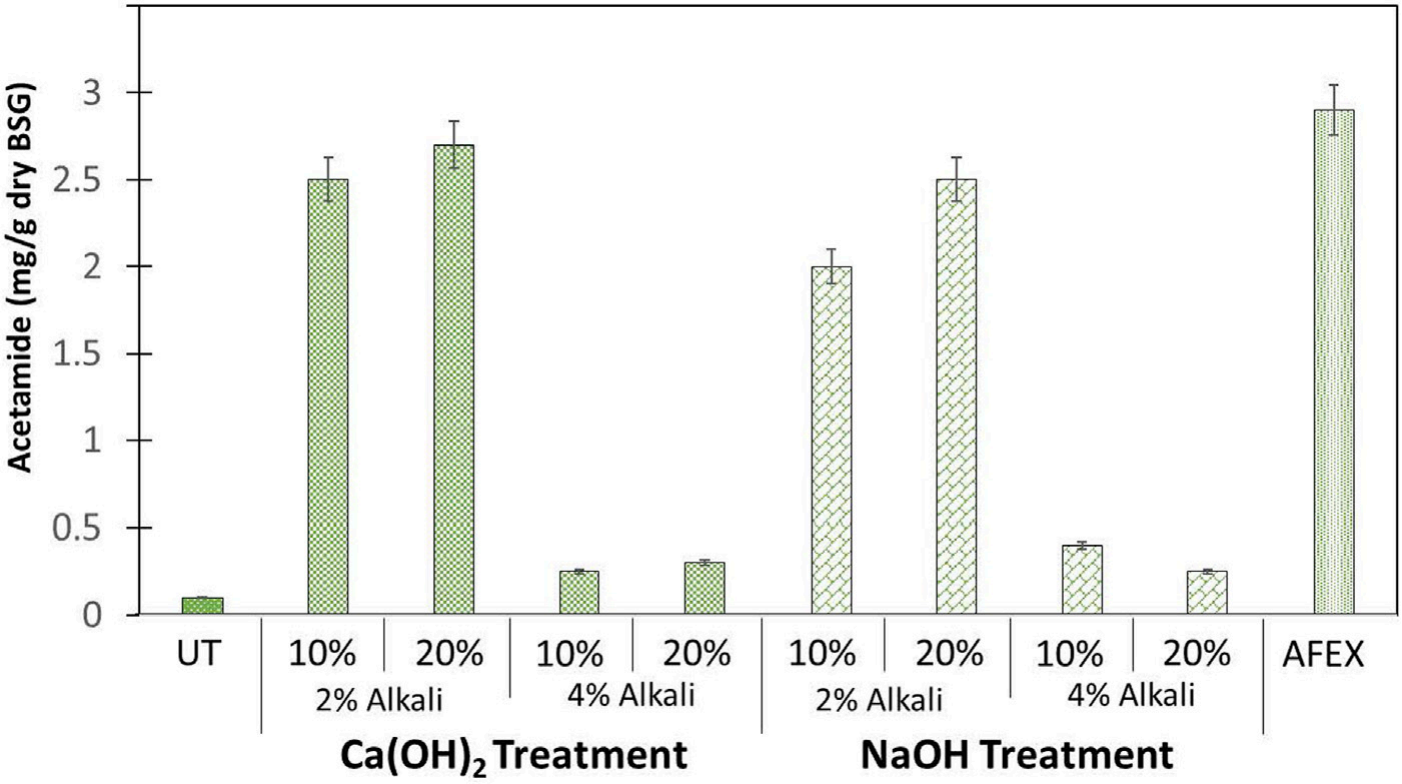
Amount of acetamide (mg/g dry weight) present in untreated (UT) BSG, AFEX pretreated BSG, and two different alkalies [NaOH and Ca(OH)_2_] treated de-esterified BSG followed by AFEX pretreatment. Here, two different solids loading (10 and 20%) and two different alkali concentrations (2 and 4%) were used during de-esterification of BSG prior to AFEX pretreatment. Error bars represent the standard deviation of two replicated experimental points.

Pretreatment of BSG using AFEX alone produced acetamide at about 3 mg/g DW BSG. We compared these results with BSG treated using mild alkali (Ca(OH)_2_) at two different concentrations (2 and 4%) and at two different solid loadings (10 and 20%) followed by carrying out AFEX. At 2% Ca(OH)_2_ treatment followed by AFEX, the concentration of acetamide significantly increased when compared to untreated BSG. We also observed that 10% solids loading produced lesser acetamide (2.5 mg/g DW BSG) when compared to 20% solids loading (2.8 mg/g DW BSG). On the contrary, when the concentration of Ca(OH)_2_ was increased from 2 to 4% followed by AFEX, we observed a significant drop (down to 0.3–0.4 mg/g DW BSG) in acetamide concentration both at 10 and 20% solids loading (Figure 5).

Treatment of BSG with strong alkali (NaOH) followed by AFEX produced a similar concentration to acetamide as mild alkali (Ca(OH)_2_) treatment followed by AFEX. We only saw a slight drop in the concentration of acetamide (2–2.5 mg/g DW BSG) at 2% NaOH treatment followed by AFEX, when compared to the concentration of acetamide (2.5–2.8 mg/g DW BSG) following treatment with 2% [Ca(OH)_2_]. Further increasing alkali concentration of NaOH from 4 to 8% at 10% BSG loading gave acetamide concentration (0.02–0.04 mg/g DW biomass) like untreated biomass (data not shown). However, considering the de-esterification efficiency and subsequent feed quality, high alkali dosage (4% alkali) and 10% TS are considered as optimum conditions while treating DDGS.

### De-Acetylation and Ammoniation of DDGS

**FIGURE 6:**
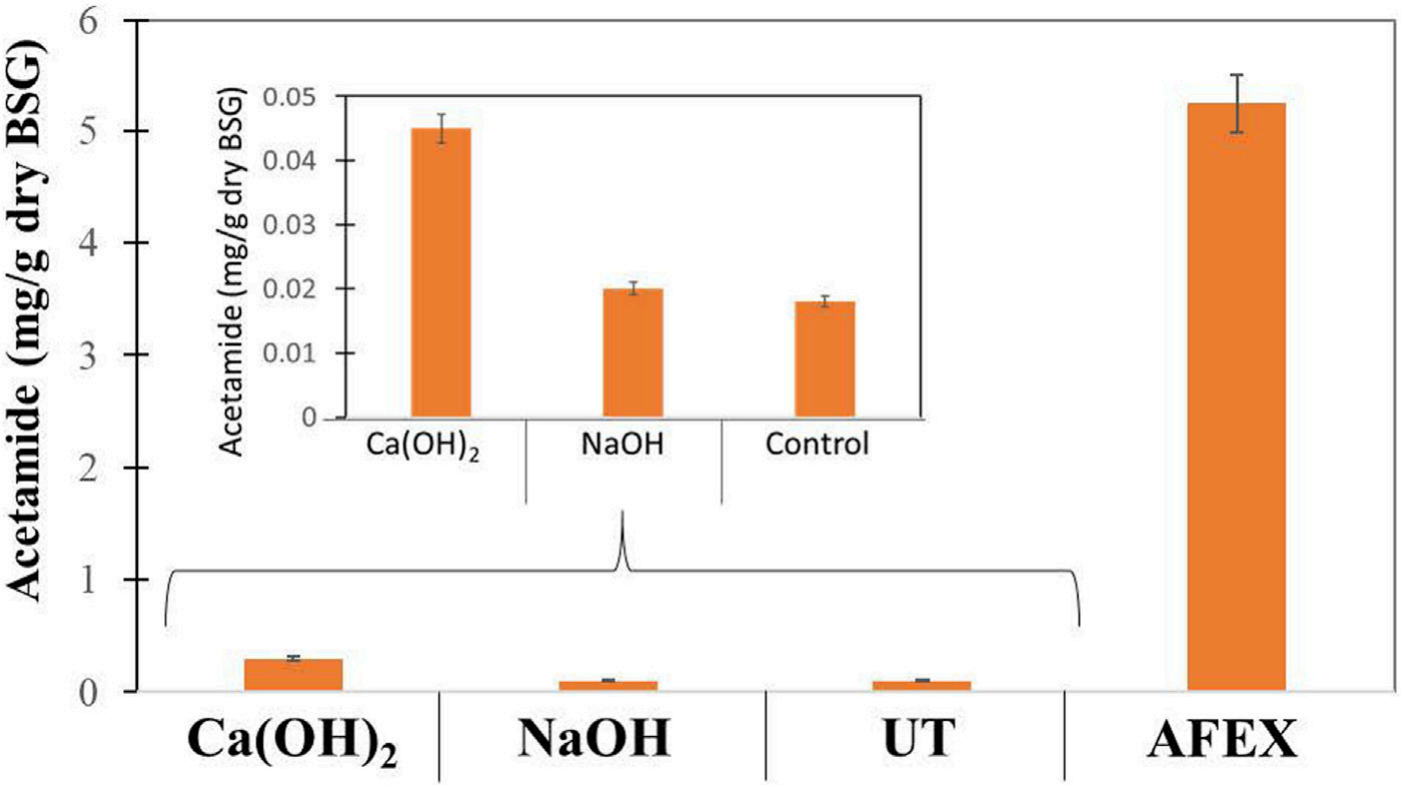
Amount of acetamide (mg/g dry weight) produced in untreated (UT) DDGS, AFEX pretreated DDGS, and two different alkalies [NaOH and Ca(OH)_2_] treated de-esterified DDGS followed by AFEX pretreatment. About 10% solids loading and 4% alkali concentration were used during de-esterification of DDGS prior to AFEX pretreatment. Error bars represent the standard deviation of two replicated experimental points.

Pretreatment of DDGS using AFEX alone produced about 5.3 mg/g DW of acetamide, which was much higher when compared to BSG, indicating that DDGS had high acetylated xylan. We compared these results with untreated DDGS and pretreated DDGS using mild alkali (Ca(OH)_2_) and strong alkali (NaOH) at 2 and 4% concentrations and at 10 and 20% solids loading followed by carrying out AFEX. When compared to 2% alkali loading, 4% alkali loading at both solids loading followed by heating at 80°C for 15 min resulted in better cleavage of ester linkages. These results show a critical concentration of alkali needs to efficiently de-esterify the BSG. Subjecting the de-esterified biomass at 4% alkali followed by AFEX produced acetamide concentrations like untreated DDGS (Figure 6). By comparing de-acetylation reactions of DDGS and BGC, we conclude that alkali treatment was more effective in the case of DDGS when compared to BSG, indicating the complexity of two different substrates for use as animal feed.

We also evaluated the total nitrogen contents of the biomass in BSG and DDGS after AFEX treatment. We found 55–57 mg/g for AFEX pretreated BSG and 72 mg/g for AFEX treated DDGS (results not shown). The more nitrogen content implies more ammoniation and de-acetylation during the AFEX treatment process that led to acetamide formation in DDGS as explained before. We also did not find a significant change in acetamide content (as total nitrogen) when varying temperature or ammonia to biomass ratio. It is important to note that AFEX pretreatment at low temperature and low ammonia to biomass ratio will be more cost-effective.

### Effect of Alkali and AFEX Pretreatment on Digestibility of BSG and DDGS

**FIGURE 7:**
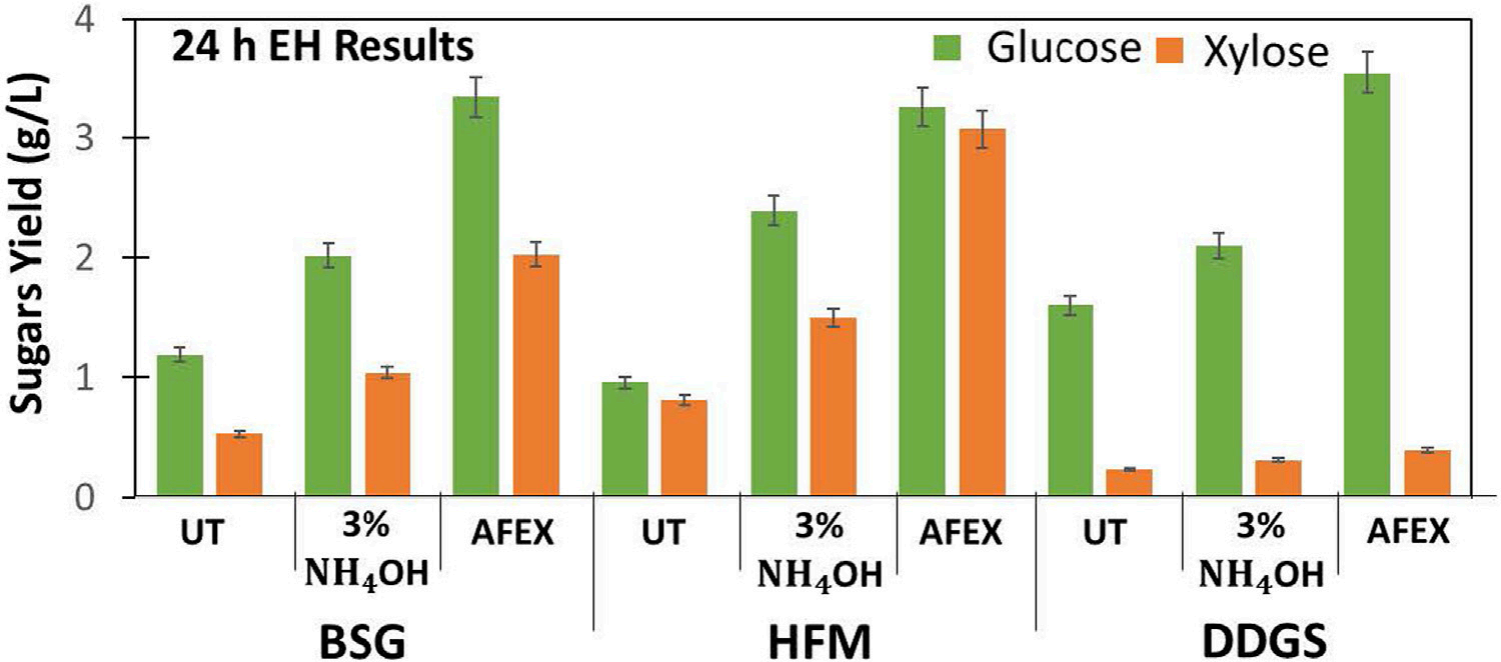
Enzyme hydrolysis of untreated (UT), 3% dilute ammonium hydroxide, and AFEX pretreated BSG, HFM, and DDGS. Green bars represent glucose, and orange bars represent xylose yield (g/L). Error bars represent the standard deviation of two replicated experimental points.

We evaluated the digestibility of untreated and two different ammonias treated (3% ammonium hydroxide and 1:1 ammonia to biomass ratio in AFEX) BSG, HFM, and DDGS using commercial enzymes (Ctec2: Htec2: Pectinex at 80:15:5 ratio). The results show that untreated BSG, HFM, and DDGS were recalcitrant and produced lower sugar yields when compared to ammonia-treated BSG, HFM, and DDGS, respectively. De-esterification of BSG, HFM, and DDG using NaOH (using 4% alkali) followed by AFEX resulted in higher sugar yield (Figure 7). Higher concentration of ammonia used during AFEX pretreatment (1:1 ammonia to biomass loading) helped to solubilize lignin and sugar oligomers and re-deposit on the surface when ammonia is released during the de-pressurizing step, thereby creating pores in biomass. The pores created in the biomass facilitate easy access of enzymes during hydrolysis by producing higher sugar conversions, as reported earlier (Chundawat et al., 2011). For BSG, we observed that 3% ammonium hydroxide treatment produced twofold higher sugar yield, and AFEX treatment produced threefold sugar yield when compared to control. These results show that the concentration of ammonia and pressure exerted during AFEX pretreatment plays an important role in increasing the digestibility of cellulose and hemicellulose. A similar increase in cellulose and hemicellulose conversation has been reported for different AFEX pretreated monocot and dicot lignocellulosic biomasses (corn stover, agave bagasse, agave leaf matter, switchgrass, reed canary grass, elephant grass, alfaalfa, miscanthus, rice straw, sorghum, sugarcane bagasse, sugarcane leaf matter, poplar) (Balan et al., 2011, 2012; Flores-Gómez et al., 2018; Mokomele et al., 2018) (Table 1).

In the case of DDGS, we observed that 3% ammonium hydroxide gave a moderate increase in glucose yield, while AFEX treatment resulted in a threefold higher glucose yield. Though we found higher glucose yield after AFEX treatment when compared to untreated DDGS, the xylose yield was still low. It has been reported that fibers in DDGS have complex xylan linkages and require specialized enzymes, such as pectinase and esterase in the cocktail, to get a higher sugar yield (Dien et al., 2008). In this work, we have demonstrated a two-step process of producing highly digestible BSG, HFM, and DDGS without any acetamide formation for the first time. We hypothesize that similar results could be obtained when two-step processing is applied to other agricultural residues. However, the processing conditions must be optimized.

### Economic Benefit of the Proposed Process

As the demand for animal feed increases, technologies need to be developed to efficiently use existing feedstocks or find additional feed resources and upgrade them to meet the nutritional requirement of animals. BSG is a sustainable feedstock available throughout the year that can be fractionated and upgraded for sale into animal feed markets. The average price of BSG and DDGS is $100–$200 and $178/dry ton, respectively (Table 3). Other comparable animal feed products in the market include corn gluten meal and wheat middling, whose prices are $125 and $168, respectively. Producing highly digestible pretreated BSG or HFM is expected to fetch a high market price. More importantly, the highly digestible animal feed will result in efficient feed utilization and less animal waste, benefiting the environment. This allows farmers to comply with the ever-tightening regulatory requirements set by the environment protection agencies. Extension of the technology to other agricultural byproducts will enhance animal feed safety and sustainability. Eventually, developing such technology will bring sustainable business opportunities for feed manufacturing companies and animal feed distributors and benefit several ruminant animal farmers around the world. The potential US market for highly digestible BSG, HFM, and DDGS is given in Table 4.

**TABLE 3:** BSG, HFM, and competitive fiber energy feed. Compositional analysis is provided based on dry matter basis.

Composition/price	BSG	HFM	Corn gluten feed	Wheat mids. (WM)	DDGS
Protein	34.0	22.0	16.5	18.0	28.0
Fat	10.0	8.0	4.5	3.5	7.0
ADF	27.0	30.0	9.9	13.0	13.0
NDF	50.0	65.0	32.0	45.0	35.0
Avg. price/dry ton	$100–200	NA	$125	$169	$178

**TABLE 4:** Potential US animal feed market for enhanced digestible BSG, HFM, and DDGS.

Species	US feed volume (million PY)	Proportion feed CPD	Average inclusion rate	Potential CPD value (TPY)
Dairy	19	0.1	0.050	95,000
Beef	21	0.1	0.050	105,000
Poultry	55	0.2	0.125	1,375,000
Swine	24	0.2	0.125	600,000
Sheep/goat	0.3	0.5	0.300	45,000
Fish	11	0.4	0.125	550,000
Companion	9	0.2	0.150	270,000
Total	—	—	—	3,040,000

CPD, crude protein degradability; TPY, ton per year.

## Conclusion

For the first time, we demonstrated a two-step pretreatment process (alkaline treatment followed by AFEX) that was an effective method for reducing or eliminating acetamide formation in lignocellulosic biomass, such as BSG, HFM, and DDGS. As detailed above, in several cases, the final acetamide content was 0.04 mg/g equal to that of untreated biomass and, therefore, safe to be used as animal feed. In addition, the pretreatment improved the digestibility by twofold to threefold when compared to untreated BSG, HFM, and DDGS. Compared to the conventional AFEX process, the amount of ammonia required to treat the biomass was lowered following calculated alkali pretreatment. This is another major advantage of the integrated process that could reduce the overall pretreatment cost of biomass without compromising the quality of the product. While only BSG, HFM, and DDGS were experimented during this study, these techniques could easily be applied to other lignocellulosic products such as corn stover, switchgrass, sugarcane bagasse, wheat straw, sorghum, energy cane, miscanthus, soybean meal, and many other widely available lignocellulosic biomasses. From here, research should focus on the optimization for each of these substrates, as well as further improvements in digestibility by adding feed enzymes commonly used in animal feed industry.

## Data Availability

The raw data supporting the conclusion of this article will be made available by the authors without undue reservation.

## Abbreviations

AFEX, ammonia fiber expansion; AraF, arabino-furanosyl; BSG,b rewer’s spent grains; CS, corn stover; DDGS, dry distillers grains with soluble; DFA, dehydro di-ferulic acids; EA, extractive ammonia; FA, ferulic acid; GAX, glucuronoarabinoxylan; HFM, high-fiber meal produced from BSG; LCC, lignin-carbohydrate complex; pCA, p-coumaric acid; UT, untreated.
